# Molecular programs induced by heat acclimation confer neuroprotection against TBI and hypoxic insults via cross-tolerance mechanisms

**DOI:** 10.3389/fnins.2015.00256

**Published:** 2015-07-28

**Authors:** Michal Horowitz, Gali Umschweif, Assaf Yacobi, Esther Shohami

**Affiliations:** ^1^Laboratory of Environmental Physiology, The Hebrew UniversityJerusalem, Israel; ^2^Department of Pharmacology, The Hebrew UniversityJerusalem, Israel

**Keywords:** traumatic brain injury and neuroprotection, heat acclimation and cross tolerance, angiotensin AT2 receptor and neurogenesis, AKT-HIF-1 signaling, hypoxia, NMDA and AMPA receptors

## Abstract

Neuroprotection following prolonged exposure to high ambient temperatures (heat acclimation HA) develops via altered molecular programs such as cross-tolerance Heat Acclimation-Neuroprotection Cross-Tolerance (HANCT). The mechanisms underlying cross-tolerance depend on enhanced “on-demand” protective pathways evolving during acclimation. The protection achieved is long lasting and limits the need for *de novo* recruitment of cytoprotective pathways upon exposure to novel stressors. Using mouse and rat acclimated phenotypes, we will focus on the impact of heat acclimation on Angiotensin II-AT2 receptors in neurogenesis and on HIF-1 as key mediators in spontaneous recovery and HANCT after traumatic brain injury (TBI). The neuroprotective consequences of heat acclimation on NMDA and AMPA receptors will be discussed using the global hypoxia model. A behavioral-molecular link will be crystallized. The differences between HANCT and consensus preconditioning will be reviewed.

## Cross-tolerance

Cross-tolerance is the phenomenon whereby exposure to one stressor induces protection from a novel stressor and such cross reinforcement raises the possibility of inducing protection to a stressor without prior exposure to it (Horowitz et al., 2004). It is an important feature of many cellular protective mechanisms and involves the plasticity of interactions of environmental-stressors at one end of the spectrum, to drugs and pharmacological interactions at the other. In this review we will focus on how adjustment to one environmental stressor limits damage caused by exposure to novel stressors i.e., heat-acclimation mediated neuroprotection cross-tolerance (HANCT).

### Heat acclimation

Heat acclimation is the term used to describe the processes occurring when an organism is exposed to environmental heat for a prolonged period. The changes at all levels of body organization lead to a new homeostasis which is achieved by the reprogramming of gene expression.

Heat acclimation (Horowitz, 2014) is achieved by persistent exposure of an organism to an ambient temperature above its normothermic range. Heat acclimation is a “within lifetime” phenotypic adaptation involving adjustments at all levels of body organization to enhance thermo-tolerance and heat endurance. The physiological criteria for the heat acclimated phenotype are reduced basal metabolic and heart rates as well as basal body temperature, concurrent with lower temperature-threshold of activation of the heat dissipation effectors. An important additional criterion is delayed thermal injuries, due to an elevated injury temperature threshold. Reprogramming of gene expression and post-transcriptional regulatory mechanisms are essential components of the acclimated phenotype. Major molecular players in the induction of heat acclimation are the Heat Shock Factor 1–Heat Shock Proteins (HSF1–HSPs) cascade and hypoxia inducible transcription factor α (HIF-1α) and its targets (Maloyan et al., 1999, 2005; Shein et al., 2005; Horowitz, 2010; Horowitz and Assadi, 2010). The 1 month needed to induce acclimatory homeostasis augments HSP72 reserves and leads to constitutive elevation of HIF-1α under normoxic environmental conditions. This implies that in the acclimated phenotype, cytoprotection can be accomplished without *de novo* HSP synthesis or abolishment of HIF-1α degradation.

Classical preconditioning (cPC) is a cellular-molecular protective mechanism evoked following exposure to a sub-lethal stress. Initially rapid, transient salvage kinases are activated followed by transcription and translation of cytoprotective proteins. Protection from subsequent exposure to the same stress lasts for 24–48 h.

In cPC, cytoproective molecular reserves are augmented via subjection to intermittent short sub-lethal stresses (i.e., thermal, ischemic, hypoxic), which confer protection to larger stressors, mostly of the same type, when given within 24–48 h (Murry et al., 1986; Obrenovitch, 2008; Thompson et al., 2013). Unlike HA (Section Preconditioning vs. Heat Acclimation Mediated Cross-tolerance below), the short-term sub-lethal stresses of cPC are insufficient to confer long-term protection even though stress-associated genes are immediate “on-demand” responders [namely, induced by preconditioning (or adaptive processes) and remain on “stand by”]. Gene chip bioinformatic analyses of the acclimatome (Tetievsky et al., 2014) showed that heat acclimation induces qualitative changes in molecules such as ion channels, receptor properties and various metabolic pathways, which can also be neuroprotective. In the frontal cortex and the hippocampus, Yacobi et al. (2014) reported significant changes in the properties of the N-methyl-D-aspartate receptor (MNDA-R) and α-amino-3-hydroxy-5-methyl-4-isoazole-propionic acid receptor (AMPA-R), while Schwimmer et al. (2006) provided evidence of changes in cytokines and various pyrogenic neuropeptides and neuropeptide receptors, as well as changes in the angiotensin AT1 and AT2 receptor ratio. These phenotypic changes were found to be neuroprotective, either by “preventive” mechanisms, which attenuate the severity of the damage, or by inducing spontaneous recovery via cytoprotective pathways and are discussed below regarding traumatic brain injury (TBI) and hypoxic stress. For details regarding HANCT following hyperoxic insults the readers are referred to Arieli et al. (2003).

### Preconditioning vs. heat acclimation mediated cross-tolerance

cPC was first reported in 1986, when Murry et al. (1986) discovered that intermittent brief sub-lethal ischemic episodes protect the heart from a subsequent sustained ischemic insult. That phenomenon, defined as “preconditioning” suggested that in cardiac patients, the multiple episodes of angina that often precede myocardial infarction actually delay cell death after coronary occlusion allowing larger areas of the myocardium to survive and function. Less than 10 years later, this preconditioning effect became widely recognized in brain research as a powerful cytoprotective tool and provided an innovative approach for the development of protective strategies (Obrenovitch, 2008). Preconditioning is a part of the universal rapid cellular adaptive mechanisms induced by many physiological and pharmacological stressors (e.g., hypoxia, ischemia, heat stress, anesthetics).

In view of the large number of inducible stress genes responding to heat exposure and/or heat acclimation (Horowitz et al., 2004; Schwimmer et al., 2004; Horowitz, 2014) we postulated that the induction of the consensus heat acclimation players, HSH1-HSP cascade or HIF-1α and targets only is unlikely to be sufficient to confer heat-acclimation-induced cross-tolerance. Gene-cluster analysis of cDNA Atlas arrays of genes representing homeostatic responses and stress-associated genes in the heart (Horowitz et al., 2004), respectively, showed a divergence between genes responding to heat stress and those responding to ischemia/reperfusion insult. The data suggest that “shared signaling cascades” are the underlying mechanism of cross-tolerance *by interacting with those involved in stress-specific or organ specific responses*.

Conceptually, both heat acclimation mediated cross-tolerance and cPC rely on transcriptional activation. However, in contrast to cPC, heat acclimation for 2 days (when apparent acclimation depends on neural activity and cPC is at its peak) impedes tolerance to novel stressors despite the rapid adaptive responses, and this is probably related to phosphorylation processes (detailed in Assayag et al., 2012). Therefore it seems that heat acclimation mediated cross-tolerance depends on long-term translational processes, which establish reserves of cytoprotective proteins (e.g., HSP72, Section Heat Acclimation above) and/or adjust electron transfer and ROS production in the mitochondria (Assayag et al., 2012). Therefore, cross-tolerance can only be tested after acclimatory homeostasis has been achieved. Recent studies from our laboratory (Treinin et al., 2003; Horowitz and Alxander-Shani, 2015) demonstrated that (i) HSF1-HSP72 cascade (at least in the heart and in *C. elegans*) is regulated in a HIF-1α independent manner and that (ii) HSP72 is essential but insufficient to confer protection in our models. It remains unclear whether HIF-1α transcriptional activation alone is sufficient to confer protection.

## Traumatic brain injury

### Epidemiology

TBI affecting about 10 million people worldwide every year, is a leading cause of death and disability and is a major social, economic and health problem (Maas et al., 2008). The World Health Organization estimates that by the year 2020 TBI will become a more common cause of death than other major diseases (Zitnay, 2005). The majority of TBI occurs among young children (0–4 years) and in the 15–45 age group, and 75% of those affected are male (Bruns and Hauser, 2003; Rutland-Brown et al., 2006; Tagliaferri et al., 2006; Maas et al., 2008). Causes of TBI differ between the age groups. In young children and the elderly, falls are the primary cause of head injury (Bruns and Hauser, 2003). Young adults are more susceptible to TBI through road traffic accidents, fights and contact sports (hockey, football, soccer) (Finfer and Cohen, 2001; Mock et al., 2004). Recently attention has been paid to military personnel suffering from TBI from blast waves (Taber et al., 2006; Hampton, 2011). Almost half of the TBI patients suffer from long-term disabilities such as neurological disorders (e.g., epilepsy and sleep disorders), neurodegenerative and psychiatric diseases, neuroendocrine disorders, and non-neurological disorders (Masel and DeWitt, 2010). In spite of extensive research no effective pharmacological intervention to facilitate recovery after TBI has been found.

### Early harmful events

The primary injury in TBI is due to mechanical forces on the brain and is accompanied by shearing and tearing of the tissues and blood vessels which trigger immediate and long-term changes in ionic homeostasis, brain metabolism and function. Following the mechanical impact, cellular/molecular mechanisms are set in motion. Many of these mechanisms, such as glutamate-induced excitotoxicity, impaired energy metabolism, oxidative stress and inflammation, are harmful and lead to neuronal cell death, tissue necrosis and functional impairments (Miller, 1993; Werner and Engelhard, 2007; de Lanerolle et al., 2015). Brain hypoxia is one of the most common secondary insults occurring following severe TBI (Chesnut et al., 1993; Jeremitsky et al., 2003). It can be initiated by TBI-induced cerebral hypo-perfusion, or apnea and hypo-ventilation mostly related to brainstem injury (McHugh et al., 2007). Yan et al. (2011) used a combined model of diffused TBI and hypoxia and showed that cerebral hypoxia exacerbates the secondary brain damage following TBI. In their studies Yan et al. (2011) demonstrated increased motor and behavioral deficits along with greater production of pro-inflammatory cytokines and sustained metabolic depression, when compared to TBI alone.

Among the early harmful events that contribute to the pathogenesis of TBI is excessive production of reactive oxygen species (ROS) and NO. The resulting reactive nitrogen species are detected minutes to hours after the insult. Brain tissue is the most vulnerable tissue to oxidative damage due to its high rate of oxidative metabolic activity, intensive production of reactive oxygen metabolites, and relatively low antioxidant capacity (for review e.g., Chong et al., 2005). The brain also contains high levels of transition metals, such as iron, which can catalyze the production of highly toxic radicals via the metal-mediated Haber–Weiss reaction (Halliwell and Gutteridge, 1989). In order to cope with oxidative stress living cells have developed several lines of defense, including antioxidative enzymes and low molecular weight antioxidants.

### Endogenous neuroprotective mechanisms

In parallel to the harmful cascades, TBI also induces endogenous neuroprotective mechanisms. The ability of cells, tissues and organisms to utilize adaptive self-protective mechanisms is now well recognized in the post-injury phase. The final outcome of TBI is determined by the balance between injury and repair mechanisms (Neary, 2005). Numerous studies have demonstrated the profound protective effect of preconditioning by brief ischemic or thermal exposures on the outcome of ischemic brain injury. The beneficial consequences of such procedures have been demonstrated in several *in vitro* (Liu et al., 2000) and *in vivo* models (e.g., Dirnagl et al., 2003; Glantz et al., 2005; Blanco et al., 2006).

As mentioned above, the functional outcome after TBI depends on the balance between the deleterious and protective mechanisms. Exposure to pre-conditioning stimuli may either inhibit key pathways in harmful cascades or activate key pathways in protective cascades. Identification of these mechanisms may facilitate the design of novel drugs that mimic the self-protective capacity of the brain.

## Molecular mechanisms underlying HA mediated neuroprotection after TBI-inherent spontaneous recovery pathways

The cross-tolerance that HA confers against TBI was investigated extensively by combining HA model (Horowitz, 1976; Maloyan et al., 2005; Tetievsky et al., 2008) with the closed head injury experimental model of TBI (Chen et al., 1996; Flierl et al., 2009). We found that functional recovery of HA rats and mice was greater, with less brain tissue damage, compared to non-HA control animals (Shohami et al., 1994a,b, 1997; Shein et al., 2007). The natural healing processes after TBI are poorly understood, therefore the enhancement of the healing and significantly improved outcomes following TBI after HA prompted us to explore the mechanisms by which HA confers neuroprotection. The accelerated healing of the injured brain in acclimated animals is unusual and provides important information regarding key inherent neuroprotective pathways.

### The importance of akt signaling

HANCT confers prolong improved recovery after TBI persisting for at least 6 weeks after injury. This is shown by enhanced motor function, improved cognitive function and reduced edema formation (Shein et al., 2005, 2007; Umschweif et al., 2010, 2013). In order to understand this protection we focused here on Akt (also known as protein kinase B), a master-regulator of pro-survival pathways in many tissues including the brain. Akt activation is enhanced following HA. Moreover, pharmacological inhibition of Akt phosphorylation abolished the beneficial effects of HA in injured mice (Shein et al., 2007), highlighting the importance of this factor in HANCT. Akt activation attenuates the intrinsic apoptosis pathway, which usually causes extensive cell death in neurons (Zhang et al., 2005). Post-injury lesion volume was smaller in the HA group compared with normothermic control mice, possibly due to inhibition of the intrinsic apoptosis pathway (Umschweif et al., 2010). Akt promotes cellular survival and healing by direct regulation of a variety cellular factors including hypoxia inducible factor 1 alpha (HIF-1α).

#### HIF-1α—a key mediator of HA mediated neuroprotection

HIF-1α is the regulatory subunit of the heterodimer HIF-1, a well-known hypoxia induced transcription factor. This subunit responds to many cellular events including hypoxia and is induced by reduced cellular oxygen availability (Singh et al., 2012). The active transcription factor recognizes and binds to the hypoxia-response elements and activates transcription of its target genes, allowing tissue adaptation to hypoxia and ischemia. Secondary ischemia due to reduced oxygen delivery to the injured tissue occurs following TBI; hence HIF-1 activation shortly after TBI fights secondary ischemia and reduces tissue loss. Interestingly, HIF-1α levels are high in HA mice and remain elevated after injury (Shein et al., 2005; Umschweif et al., 2013). The beneficial effect of elevated HIF-1α was lost after inhibition of HIF-1 dimerization with acriflavine (Umschweif et al., 2013). Acriflavine eliminated the spontaneous recovery after TBI and in the first days after TBI no improvement in motor ability was noted, confirming the role of HIF-1α in mediating neuroprotection. Furthermore, in HA mice HIF-1 inhibition not only prevented the enhanced recovery compared to normothermic mice, but motor ability deteriorated, lesion volume was greater as was the death rate of the injured mice (Umschweif et al., 2013). This highlights the importance of HIF-1 in both spontaneous recovery and HANCT. The molecular mechanisms underlying the contribution of HIF-1 to HA mediated neuroprotection are yet to be elucidated, however, there is an evidence that may illuminate events downstream to HIF-1.

Among the cytoprotective and angiogenesis related genes that are upregulated by active HIF-1 is vascular endothelial growth factor (VEGF). VEGF has been described as a potent inducer of neuroprotection and neurogenesis after TBI (Thau-Zuchman et al., 2010, 2012). Therefore it is possible that some of the beneficial effects of HIF-1 in HA mice may be due to the upregulation of VEGF. HA increases VEGF levels and these remain high after TBI. On the other hand, VEGF levels are significantly attenuated following inhibition of HIF-1. It is therefore reasonable to speculate that VEGF is one of the downstream targets mediating HIF-1 induced neuroprotection in HA mice.

Another HIF-1 target gene that may facilitate this effect is the glucose transporter 1 (GLUT1) which is abundant in the brain. Similarly to VEGF, GLUT1 is upregulated in HA brains and this effect is eliminated by HIF-1 inhibition (Umschweif et al., 2013). GLUT1 induction following TBI potentially allows more efficient glucose uptake by brain cells and may fight the energy depletion that results from secondary ischemia. Notably, we demonstrated that HA induces a significant concomitant increase in the expression of nuclear HIF-1α and EpoR prior to and post TBI suggesting the involvement of this pathway in HA-induced neuroprotection (Shein et al., 2005).

Collectively, HIF-1 is a key mediator of HA mediated neuroprotection and elevated HIF-1 levels contribute to the improved outcome of acclimated animals after TBI.

### Angiotensin receptor type 2: an upstream regulator of neuroprotection and neurorepair in injured HA mice

The crucial role of HIF-1 activation in HA mediated neuroprotection has placed HIF-1 in the spotlight in the search for a new drug target which will successfully mimic the protective effects of HA on TBI. Pharmacological stabilization of HIF-1 independent of cellular oxygen levels may occur following activation of certain growth factor receptors such as insulin like growth factor or epidermal growth-factor (PMID:13130303), which are relevant in the fight against cancer. A different upstream receptor activates HIF-1 in the brain, the angiotensin receptor type 2 (AT2).

AT2 is abundant in the brain during development and under stress (Dumont et al., 1999; Guimond and Gallo-Payet, 2012) including after brain ischemia. It was also elevated in rats following HA (Schwimmer et al., 2004). AT2 activation improves the outcome following brain ischemia and reduces lesion volume (McCarthy et al., 2009, 2012; Guimond and Gallo-Payet, 2012). Taken together, these data promote the hypothesis that increased AT2 levels in the HA phenotype facilitate neuroprotection and improved TBI outcome. Interestingly, AT2 levels were not elevated in HA mice; however, pharmacological blocking of AT2 by a specific antagonist (PD123319) abolished the beneficial effects of HA. HA was found to affect AT2 signaling by increasing levels of another receptor which transactivates AT2, the nerve growth factor receptor, TrkA. HA increased levels of TrkA as well as its endogenous ligand, nerve growth factor (NGF). These events, in combination with the induction of brain derived neurotrophic factor (BDNF) and its receptor TrkB seen in HA mice, may explain the extreme effects of AT2 blocking on TBI outcome. Since both NGF and BDNF increase Akt phosphorylation and in turn activate HIF-1, blocking AT2 inhibits these signals which contribute to neuroprotection in HA mice. Indeed, by blocking AT2 levels in HA mice the levels of NGF and BDNF as well as HIF-1α levels were attenuated. This may explain why the detrimental effects of AT2 blocking were only noted in HA mice, and hardly affected the recovery of control mice after injury. An interesting observation of AT2 blocking in HA mice is the effect on induced neurogenesis seen in HA mice after TBI. HA not only led to TBI induced cell proliferation, but also induced neurogenesis after injury, as seen in the SVZ, the dentate gyrus and in the injury region. These neuroregenerative events are orchestrated and triggered by AT2 signaling—NGF, BDNF, and Akt and were eliminated by blocking AT2, thus, supporting the notion of AT2 as modulator of neurorepair (Umschweif et al., 2014a).

#### AT2 activation after TBI mimics the beneficial effects of long term heat acclimation prior to injury

After revealing the role of AT2 signaling and identifying it as an indispensable factor in HA-mediated neuroprotection, we examined whether these benefits could be mimicked by direct activation of AT2 after TBI.

Using a specific AT2 agonist (CGP42112A), administered for 3 days following TBI we obtained an effect similar to that achieved by HA. The recovery of motor function of the agonist treated mice was somewhat delayed, beginning about 2 weeks after TBI and significantly progressing over the next 3 weeks. Moreover, cognitive function assessed by the ability of the injured mice to recognize a novel object also improved in a dose dependent manner. Lesion volume in the agonist treated mice was smaller than that in the control group (Umschweif et al., 2014b). The molecular mechanism underlying these effects was confirmed. Neurotrophins (NGF, BDNF) and their downstream kinases—Akt and Erk1/2 were induced by the AT2 agonist and blocking the receptor inhibited these molecular effects. These early protective events, occurring within 24 h of the injury, seem to set the stage and probably initiated the neurorepair events seen for weeks after injury. Early AT2 activation induced proliferation and neurogenesis in the neurogenic niches. Interestingly, around the injury site the elevated number of newborn neurons was at the expense of newborn astrocytes (Umschweif et al., 2014b).

To date the molecular mechanisms which were found to underlie the neuroprotective and healing effects of HA, involve Akt, HIF-1, GLUT1, VEGF, NGF, BDNF, and Erk1/2. Pharmacological activation of AT2 after injury partially mimics the beneficial effects of pre-injury HA, and affects all these targets.

## Hypoxia from low PO_2_ partial pressure: HA mediated neuroprotection via NMDA-R and AMPA-R remodeling-injury “Prevention”

The most sensitive cells to hypoxic/ischemic stress are the neurons of the cornus ammonis 1 (CA1) hippocampal layer and those of layers 3–6 of the frontal cortex (Kirino et al., 1984). Following exposure to hypoxic/ischemic stress there is a massive glutamate discharge which over-activates glutamate-gated ion channels (Choi, 1992; Doble, 1999) and kills these neurons. This is mediated via NMDA-excitotoxicity, free radical formation, lactic acidosis, and inhibition of protein synthesis (Gozal et al., 1998; Lau and Tymianski, 2010). Cell death starts 2–3 days after insult and may continue for several weeks. It is probably caused by calcium influx via glutamate-gated channels during the first hours after the stress (Bochet et al., 1994; Jonas et al., 1994; Osuga and Hakim, 1994; Nadler et al., 1995; Kaur et al., 2012).

In contrast to the previous sections of this review where the impact of heat acclimation on spontaneous recovery signals induced post TBI were discussed, in this section we will describe a hypoxia rat model, which is achieved by exposure of the animals to a mixture of air and nitrogen adjusted to achieve 4.5 ± 0.5% oxygen for 15 min. We will focus on how HA causes hypoxic tolerance via NMDA receptor remodeling. The involvement of NMDA-R in the process of delayed cell death after hypoxic stress is well-established, including its upregulation and increased activity in the post-stress period.

Briefly, the NMDA-R is comprised of several subunits. The GluN1 subunit is present in all NMDA-R assemblies; therefore its levels are used as a marker of NMDA-R on the external cell surface (Kutsuwada et al., 1992; Bochet et al., 1994; Monyer et al., 1994). The GluN2 subunits are located on the external cell membrane and GluN2A and GluN2B are abundant in the hippocampus and frontal cortex, the regions most susceptible to hypoxic stress (Chen et al., 1999; Erreger et al., 2005). Ion channels made of the GluN1/GluN2A combination are four times more permeable to calcium than GluN1/GluN2B. Therefore, (in brain areas where the presence of other NMDA-R subunits is negligible) when the GluN2B/GluN2A ratio is greater than 1, there is a lower channel opening probability and less calcium penetration. In congruence with clinical behavioral tests demonstrating that heat acclimated rats are more able to deal with hypoxia than non-acclimated rats, we showed: (i) that the heat acclimated group had less NMDA-Rs than controls under both basal and hypoxic conditions and (ii) an increased GluN2B/GluN2A ratio in response to hypoxia, with controls showing a reciprocal effect. Collectively, the data indicate that the protective changes in the NMDA-R against hypoxia following long-term heat acclimation are not only the result of quantitative shifts, but qualitative changes in the sub-unit profile of the receptor also occur. Similarly, AMPA-R, another important glutamate-gated ion channels that causes cell damage during hypoxia via alteration of the GluA2 subunit, is also remodeled (Gorter et al., 1997; Carriedo et al., 1998). The HA group responds to hypoxic insult with a significant increase in GluA2 protein levels in contrast to the rapid declines noted in controls. The timing of this decrease, so soon after insult, implies that a damaging rapid increase in inter-cellular calcium concentration occurs in non-acclimated rats.

## Translational benefits of heat acclimation

Studying HANCT improves our understanding of natural, intrinsic mechanisms of neuroprotection. This form of conditioning enhances innate protective mechanisms and enables the organism (or tissue) to combat future insults. HANCT *per se* is impractical as a therapeutic modality as it cannot be applied post-injury.

However the multiple spontaneous signals of recovery, which are evoked by HANCT and minimize neuronal death, can be translated into novel neuroprotective strategies. The inference (i.e., in Section AT2 Activation after TBI Mimics the Beneficial Effects of Long Term Heat Acclimation Prior to Injury above) that pharmacological activation of AT2 (Umschweif et al., 2014b) or treatment with erythropoietin (Shein et al., 2008) is neuroprotective after TBI, stems from our studies on HANCT.

Our translational approach is supported by the interesting similarities between the molecular neuroprotective mechanisms invoked by moderate hypothermia as a therapy (Florian et al., 2008; Dietrich and Bramlett, 2010; Joseph et al., 2012), representing the reciprocal edge of thermal impacts and neuroprotection. Moderate hypothermia reduces excitotoxicity by decreasing extracellular glutamate levels, furthermore apoptosis, inflammation, edema and infarct size are reduced and motor function recovery improves. Similar protective pathways are part of the neuroprotective repertoire conferred by heat acclimation in mice (Shein et al., 2007; Umschweif et al., 2010) and rats (Shohami et al., 1994a,b).

## Concluding remarks

In this review we discussed the neuroprotective properties of phenotypic adaptations to prolonged exposure to environmental heat (heat acclimation—HA) which occur via potentiation of endogenous defense mechanisms. Damage is less severe e.g. via remodeling of NMDA-R and AMPA-R subunit profiles or by attenuating injury processes by recruiting augmented “on demand” constitutive cytoprotective networks.

**Figure 1:**
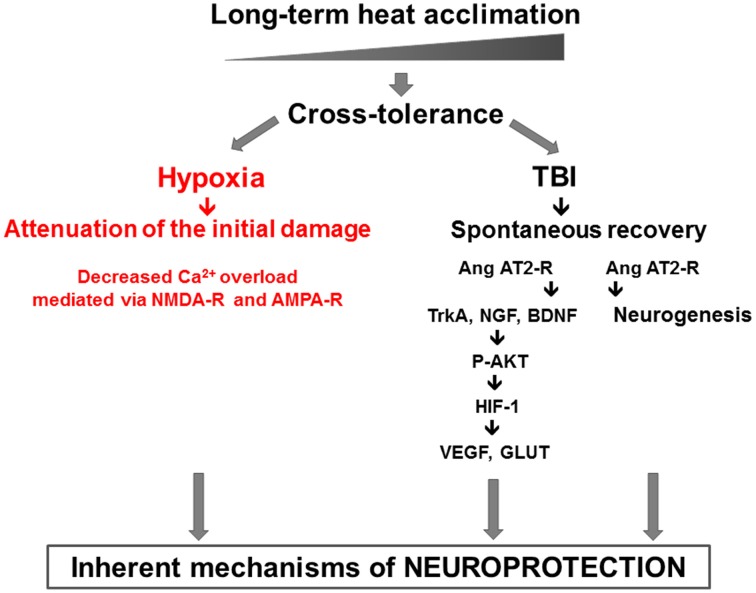
**The two pronged approach of heat acclimation mediated neuroprotection: “prevention,” or injury attenuation, shown in our hypoxia model via decreased Ca^2+^ permeability and “spontaneous recovery” shown in the TBI model**. In the latter, the role of AT2 angiotensin receptors in neurogenesis and P-AKT-HIF-1 cascade are delineated.

These are further modulated by dynamic components such as AKT-HIF-1α cascade (Akt, HIF-1, GLUT1, VEGF, NGF, BDNF, and Erk1/2). Our findings confirm the role of enhanced antioxidative, antiapoptotic, and anti-inflammatory capacities (Shein et al., 2007) in HA TBI mice. Upstream pharmacological stabilization of the HIF-1 cascade, independent of cellular oxygen levels, occurs via angiotensin receptor type 2 (AT2) was described. Furthermore, AT2 receptor activation also induces cell proliferation and neurogenesis after TBI, seen in the SVZ, the dentate gyrus and in the injury region of HA mice. *Pharmacological activation of AT2 after injury partially mimicked the beneficial effects of pre-injury HA*. The HA mediated neuroprotective features discussed are presented in Figure 1.

### Conflict of interest statement

The authors declare that the research was conducted in the absence of any commercial or financial relationships that could be construed as a potential conflict of interest.
